# Expression Analysis of Immune Related Genes Identified from the Coelomocytes of Sea Cucumber (*Apostichopus japonicus*) in Response to LPS Challenge

**DOI:** 10.3390/ijms151119472

**Published:** 2014-10-27

**Authors:** Ying Dong, Hongjuan Sun, Zunchun Zhou, Aifu Yang, Zhong Chen, Xiaoyan Guan, Shan Gao, Bai Wang, Bei Jiang, Jingwei Jiang

**Affiliations:** Liaoning Key Lab of Marine Fishery Molecular Biology, Liaoning Ocean and Fisheries Science Research Institute, Dalian 116023, China; E-Mails: ebuma@sina.com (Y.D.); hong123juan@163.com (H.S.); yangaifu@sohu.com (A.Y.); ch_zhong@163.com (Z.C.); guanxiaoyan201@163.com (X.G.); gs_7920@163.com (S.G.); wangbai1980@hotmail.com (B.W.); jiangbei1983@163.com (B.J.); weijingjiang@live.cn (J.J.)

**Keywords:** sea cucumber (*Apostichopus japonicus*), differentially expressed genes, quantitative real-time polymerase chain reaction

## Abstract

The sea cucumber (*Apostichopus japonicus*) occupies a basal position during the evolution of deuterostomes and is also an important aquaculture species. In order to identify more immune effectors, transcriptome sequencing of *A. japonicus* coelomocytes in response to lipopolysaccharide (LPS) challenge was performed using the Illumina HiSeq™ 2000 platform. One hundred and seven differentially expressed genes were selected and divided into four functional categories including pathogen recognition (25 genes), reorganization of cytoskeleton (27 genes), inflammation (41 genes) and apoptosis (14 genes). They were analyzed to elucidate the mechanisms of host-pathogen interactions and downstream signaling transduction. Quantitative real-time polymerase chain reactions (qRT-PCRs) of 10 representative genes validated the accuracy and reliability of RNA sequencing results with the correlation coefficients from 0.88 to 0.98 and *p*-value <0.05. Expression analysis of immune-related genes after LPS challenge will be useful in understanding the immune response mechanisms of *A. japonicus* against pathogen invasion and developing strategies for resistant markers selection.

## 1. Introduction

Echinoderms represent the basal deuterostomes and play important roles in evolutionary history. Studies on immune defense mechanisms and investigation of immune-related genes in echinoderms will provide insights into the immune evolution of deuterostomes. The molecular basis of echinoderm immune systems has been greatly elucidated since the publication of the sea urchin (*Strongylocentrotus purpuratus*) genome, and some of the findings have changed our paradigms about comparative immunology [1,2]. The interesting findings in sea urchins set the basis for the future studies on comparative immunology among echinoderm species. Sea cucumbers and sea urchins were assessed to diverge around 500–600 million years ago [3]. During the past decade, some immune-related genes in two important species of sea cucumbers had been analyzed. For instance, in the sea cucumber (*Holothuria glaberrima*), 22 immune putative genes were identified and among these, five genes, including *melanotransferrin*, *serum amyloid A* (*SAA*), *kazal-type serine proteinase inhibitor* (*SPI*), *α-2-macroglobulin domain* (*A2M*) and *DD104* were detected to be up-regulated after lipopolysaccharide (LPS) challenge [4,5]. Recently, many immune-related genes including the toll-like receptor (TLR) pathway molecules [6,7,8,9], complement components [10,11], heat shock proteins (Hsps) [12,13], lysozyme [14] and mannose binding lectin (MBL) [15] from echinoderms were identified and characterized. Previous studies suggested that different immune-related genes with different phylogenetic characteristics were conducive to the identification of resistant markers related to different diseases in echinoderms.

The sea cucumber (*Apostichopus japonicus*), which belongs to Holothuroidea (Echinodermata), is of great significance in evolutionary research. *Apostichopus japonicus* is also an important economic species in China with an impressive production of over 190,000 tons in 2013 [16]. However, farming diseases, especially the skin ulceration syndrome (SUS), became one of the major limiting factors in the development of the *A. japonicus* industry in recent years. It was reported that the potential pathogens causing SUS might be Gram-negative bacteria [17]. As the main component of the cell wall of Gram-negative bacteria, LPS was proved to be able to induce significant immune responses in sea cucumbers [10,11,13]. In addition, LPS can directly cause cellular injury, dysfunction and death [18,19]. Thus, assessing the immune responses to LPS that mimic Gram-negative bacteria provides rich resources to clarify immune regulation networks. As marine invertebrates, *A. japonicus* relies completely on the innate immune system to resist pathogen infection [20]. *Apostichopus japonicus* coelomocytes, probably originated from axial organ, haemal system, polian vesicles, dermal connective tissue and coelomic epithelia, are composed of a variety of morphological cell types with different immunochemical characteristics and regarded as the main immune effector cells [21,22]. Coelomocytes may infiltrate other tissues and organs to participate in immune defense reactions. In response to microbes or other foreign materials, coelomocytes undergo significant variations in the composition of subpopulations [23,24,25,26,27,28,29]. In general, the high value of *A. japonicus* in immune evolutionary research, the urgency of disease controlling in culture, and the importance of coelomocytes in immune defense motivated us to perform the analysis of immune-related genes in *A. japonicus* coelomocytes.

Based on high-throughput sequencing technology, RNA sequencing (RNA-Seq) has been shown to be an efficient way to conduct transcriptome profiling and identify differentially expressed genes (DEGs) in invertebrates for its advantages in low cost per base, high throughput, and reproducibility in dynamic expression analysis [30,31,32,33]. Till now, several transcriptome sequencing projects have been conducted on different tissues and different developmental stages of *A. japonicus* [34,35,36,37]. However, the information about immune-related genes is still limited in *A. japonicus*. Hence, the large-scale transcriptome sequencing of *A. japonicus* coelomocytes was performed to examine the expression patterns of immune-related genes after LPS challenge in this study. A total of 107 immune-related genes playing critical roles in pathogen recognition, reorganization of cytoskeleton, inflammation reactions and apoptosis were summarized to investigate the cellular immune mechanisms of sea cucumber in response to LPS challenge.

## 2. Results and Discussion

The characterization of immune-related genes and comprehensive analyses of gene expression profiles in *A. japonicus* coelomocytes based on transcriptome results are reliable. Although the heterogeneous nature of coelomocytes might result in different gene expression patterns, our primary purposes were to gain a broad understanding of coelomocytes in responses to LPS by analysis of gene expression signatures and provide early insights into important immune pathways and processes. In the previous study, we have identified 1330, 1347 and 1291 DEGs in the coelemocytes of *A. japonicus* at 4, 24 and 72 h, respectively, after LPS challenge [37]. In this study, we further investigated the DEGs involved in the immune responses from extracellular interaction with LPS to the inner nucleus activities. Based on Gene Ontology (GO) and the Kyoto Encyclopedia of Genes and Genomes (KEGG) pathway annotations, manual blast and literature searches, 107 DEGs with nonredundant (Nr) annotations were selected and divided into four main functional categories including: (1) Pathogen recognition (25 genes); (2) Reorganization of cytoskeleton (27 genes); (3) Inflammation reactions (41 genes); and (4) Apoptosis (14 genes). A subset of these candidates was listed in Table 1. There are 37, 57 and 46 significantly expressed genes at 4, 24 and 72 h, respectively. More than half of the 107 genes (61 genes) did not return back to normal expression level at 72 h. In the intestine of *Holothuria glaberrima* stimulated with LPS, the annotated sequences were classified into four functional groups including cytoskeletal proteins, metabolic enzymes, metal ion transport/metabolism and defense/recognition [38], which were identical to the annotations of DEGs from the *A. japonicus* coelomocytes transcriptome analysis. However, there is little correlation between gene expressions of the coelomocytes in sea urchin and those in sea cucumber [38], indicating that the comparison of gene expression among echinoderm species will provide new insights into the echinoderm immunity. Therefore, the discussion about the functions and classifications of different genes in echinoderms was conducted as follows.

**Table 1:** A subset of candidate genes involved in the immune response to lipopolysaccharide (LPS) challenge. Values at three time points indicate the fold changes relative to the control.

Gene Name	Transcript ID	4 h	24 h	72 h
**Pathogen Recognition**				
*CD36-like protein*	CL21862.Contig1_haishen	−11.13	1.47	−1.44
*Cytosol-type hsp70*	CL15292.Contig1_haishen	1.53	20.97	3.39
*Fucolectin-7-like*	CL7223.Contig2_haishen	13.45	−8.63	−4.76
*Fibrinogen-like protein*	CL220.Contig10_haishen	2.58	1.70	2.32
*Heat shock protein 90*	Unigene44996_haishen	2.93	4.49	6.18
*Lipopolysaccharide-binding protein*	CL17187.Contig3_haishen	1.79	−1.99	−2.75
*Mannan-binding C-type lectin*	CL3438.Contig1_haishen	1.16	−1.23	−2.35
*Scavenger receptor cysteine-rich protein type 12 precursor*	CL14054.Contig1_haishen	22.16	39.40	6.02
*Toll-like receptor*	CL791.Contig3_haishen	−1.21	2.97	1.37
*Toll-like receptor 3*	CL4619.Contig1_haishen	1.44	1.73	1.48
**Reorganization of Cytoskeleton**				
*Actin*	Unigene6143_haishen	1.92	−1.19	−1.67
*Amassin 2 precursor*	Unigene33380_haishen	−12.73	−2.31	−5.90
*Amassin 4 precursor*	Unigene32576_haishen	−5.62	−2.50	−3.51
*Focal adhesion kinase*	CL4773.Contig4_haishen	−1.30	−6.11	1.27
*Myosin V*	CL15948.Contig2_haishen	−2.04	−3.73	−11.79
**Inflammation**				
*Complement component 3*	CL9805.Contig1_haishen	−1.61	1.31	1.09
*Complement component 3-2*	Unigene40467_haishen	−1.97	1.19	10.33
*Complement factor B*	CL3046.Contig1_haishen	1.18	1.14	1.23
*Complement factor B-2*	CL3046.Contig2_haishen	−1.41	1.51	1.26
*Complement factor H-like*	CL339.Contig6_haishen	1.00	1.17	1.31
*LPS-induced TNF-α factor*	Unigene174_haishen	−1.12	1.23	1.23
*Myeloid differentiation primary response gene 88*	Unigene40451_haishen	1.18	1.11	1.32
*NF-κB transcription factor Rel*	CL9343.Contig1_haishen	1.32	1.39	1.30
*TBK1- like*	CL13483.Contig1_haishen	1.05	1.18	−1.04
*TNF receptor-associated factor 3*	CL13373.Contig2_haishen	−1.13	1.83	−1.07
*TNF receptor-associated factor 6*	CL11554.Contig2_haishen	1.49	1.56	1.12
*Thymosin β*	CL4869.Contig1_haishen	−5.82	−3.32	−1.32
**Apoptosis**				
*Caspase 6*	CL16102.Contig1_haishen	2.53	6.15	19.29
*Caspase 8*	CL18389.Contig1_haishen	−1.06	1.28	1.16
*Cathepsin B*	Unigene3030_haishen	8.86	7.67	9.38
*Lysozyme*	Unigene8800_haishen	5.13	3.56	1.85

CD36, Cluster of differentiation 36; LPS, lipopolysaccharide; TNF, tumor necrosis factor; NF-κB, nuclear factor-κ-B.

### 2.1. Pathogen Recognition

As the first line of the host immune defense system, the pattern recognition receptors (PRRs) can detect the conserved molecular signatures derived from invasive pathogens (e.g., LPS). In the pathogen recognition category, 22 of the 25 genes were highly induced, including *scavenger receptor cysteine-rich protein (SRCR) type 12 precursor* (39.4-fold), *cytosol-type Hsp70* (21-fold) and *fucolectin-7-like* (13.8-fold) (Table 1). SRCRs were first identified in macrophages and their extracellular part could directly bind polyanionic ligands like bacterial LPS [39]. A member of *SRCRs* was up-regulated significantly and continuously (about 22-fold at 4 h, 39-fold at 24 h, 6-fold at 72 h, Table 1) in *A. japonicus* coelomocytes after LPS challenge. The dynamic expression patterns and high multiple gene models of *SRCRs* in sea urchin and amphioxus confirmed their indispensible roles in host defense [40,41]. Although some fragments of *SRCRs* were found in our transcriptome result, the comparison of *SRCR* diversity between sea urchin and sea cucumber will not be meaningful and convincing unless the genome of sea cucumber is available. Cluster of differentiation 36 (CD36), a transmembrane glycoprotein from scavenger receptor class B (SRB) family, is defined as a multi-ligand scavenger receptor and greatly involved in various biological processes [42]. It has been reported that there is one *CD36* gene in human, five in sea urchin and three in amphioxus [41]. Recently, in the basal chordate amphioxus *Branchiostoma japonicum*, a CD36 homologue was identified with functions closely involved in immune defense [43]. In this study, a CD36-like gene was down-regulated (more than 11-fold, Table 1) immediately after LPS challenge. Further investigations are expected to confirm the copy numbers and specific functions of CD36-like genes in sea cucumber. Hsps also possess the LPS binding ability and the significant up-regulation of *cytosol-type Hsp70* may be caused by its interaction with LPS [44]. Under stress, the synthesis of Hsp90 increased several fold in *A. japonicus* coelomocytes [45]. Similarly, continuous up-regulations of *Hsp90* were observed after LPS challenge in this study (Table 1). Being one of the oldest PRR families, TLRs could recognize pathogen-associated molecular patterns (PAMPs) from a broad array of pathogens and play key roles in the subsequent activation of innate and adaptive immune responses in vertebrates. In *A. japonicus*, full-length cDNAs of *AjToll* and *AjTLR3* have been cloned and their expression changes after LPS challenge were moderate [8]. Some pathogen recognition genes, such as *fibrinogen-related proteins* were up-regulated (2.58-fold at 4 h, Table 1) in the coelomocytes of *A. japonicus* but not in *H. glaberrima* after LPS challenge [38]. This may be attributed to the differences in species, environment and sampling times. In order to explain the mechanisms of pathogen recognition in sea cucumbers, follow-up studies may be focused on the diversity and specificity of PRRs.

### 2.2. Reorganization of Cytoskeleton

Previous studies indicated that the populations of coelomocytes in sea cucumber varied upon stimulation with different PAMPs. Especially under LPS challenge, there is an effective increase in phagocytic activity [4,5]. These variations may be attributed to the participation of actin in forming dynamic fibrils or filaments that provide shape and mobility for coelomocytes. In this study, 27 genes were involved in cytoskeleton reorganization, of which 25 genes had marked expression variations. For example, *amassin 2 precursor* (−12.7-fold), *myosin V* (−11.8-fold) and *focal adhesion kinase* (−6.1-fold) were significantly down-regulated (Table 1). Amassin protein, containing an olfactomedin domain, was first identified from the coelomic fluid of *S. purpuratus* [46]. It functions as a mediator of cell adhesion or coelomocytes aggregation in response to injury or infection. Besides the important roles in controlling actin polymerization and permeability during pathogen infection, myosins were also involved in cytoskeleton depolymerization which led to host cell apoptosis [47,48]. The down-regulations of cytoskeleton reorganization associated genes including *amassins* and *myosin V* suggested that cytoskeleton depolymerization might facilitate pathogen invasion [49].

### 2.3. Inflammation Reactions

**Figure 1:**
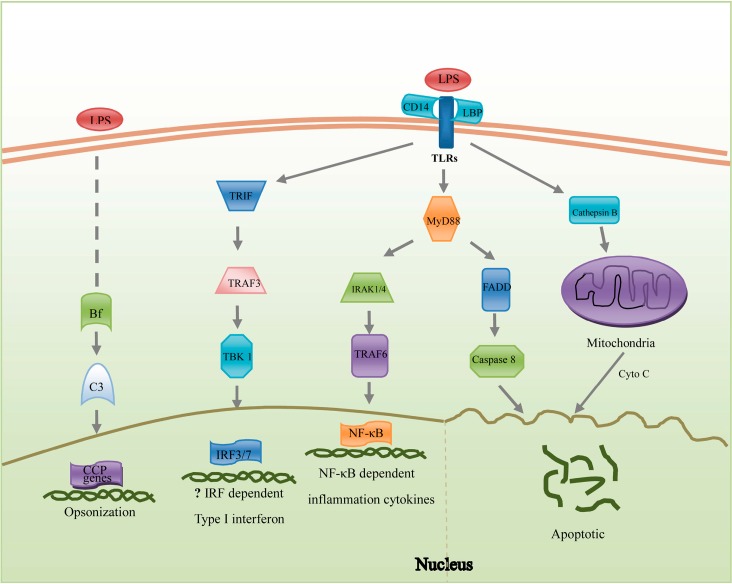
Hypothetical diagram of LPS-triggered inflammation and apoptosis pathways summarized in *A. japonicus* coelomocytes. Genes listed here play important roles in these potential pathways. On the left, three pathways will promote the expression of inflammation factors. The existence of the Type I type I interferon (IFN) pathway was unclear for the absence of IFN homologues in invertebrates. On the right, the apoptosis pathway will result in the degradation of DNA. The abbreviations: LPS, lipopolysaccharide; CCP, Complement control proteins; TRIF, TLR and interleukin-1 receptor (TIR) domain-containing adaptor inducing IFN-β; Cyto C, Cytochrome C; TRAF, tumor necrosis factor (TNF)-receptor-associated factor; TBK1, TRAF family member-associated NF-κB activator (TANK)-binding kinase 1; IRF3/7, interferon regulatory factor 3/7; CD, cluster of differentiation; LBP, lipopolysaccharide binding protein; TLR, toll-like receptor; MyD88, myeloid differentiation primary response gene 88; FADD, Fas-associated death domain protein; IRAK1/4, interleukin-1 receptor-associated kinase 1/4; NF-κB, nuclear factor-κ-B.

Genes included in inflammatory reactions are relatively abundant (41 genes) and mainly involved in the TLR signaling pathway, Type I interferon (IFN) pathway and complement pathway (Figure 1). Activated by microbial antigens, TLR pathways predominantly signal to nuclear factor-κ-B (NF-κB) by myeloid differentiation primary response gene 88 (MyD88)—Dependent pathway which leads to the release of pro-inflammatory cytokines [50]. In response to *Vibrio splendidus*, MyD88 and *TNF-receptor-associated factor 6* (*TRAF6*) were all up-regulated in *A. japonicus* [7]. Collectively, the variations of genes in the TLR signaling pathway were less significant than those in other immune pathways, which provided new paradigms in understanding of *A. japonicus* immune responses. Although some genes in the type I IFN pathway, such as TRAF3, *TRAF family member-associated NF-κB activator* (*TANK*)-*binding kinase 1* (*TBK1*) and *interferon regulatory factor 3 (IRF3)* were identified in *A. japonicus*, homologues of IFNs were not yet found. In future studies, it is worth considering whether IFNs are beneficial in bacterial infections [51].

As a central system in innate immunity, the complement system is widely distributed in deuterostomes. Among the components of the complement system, complement component 3 (C3) plays a pivotal role in the activation of classical, alternative and lectin pathways [52]. Interestingly, two different *C3* gene models were identified in the coelomocytes of sea urchin and sea cucumber, respectively [1,10]. In our transcriptome result, both of the *C3* genes were all down-regulated at 4 h, and then *C3-2* was significantly up-regulated at 72 h while *C3* was recovered to the initial expression level. As the second complement component in the alternative pathway, complement factor B (Bf) can activate *C3* and opsonize foreign cells to enhance host phagocytosis and subsequent destruction [53]. Until now, two *Bf* cDNA sequences (*complement factor B* and *B-2*) with high similarity and different expression patterns have been confirmed in *A. japonicus* [11] and their transcripts were moderate at all examined time points (Table 1). Complement factor H (*CFH*) was involved in the regulating of the alternative complement pathway in *S. purpuratus* and *A. japonicus*. Because of the repetitive structure, it is difficult to count the gene number of *CFH*. The polymorphisms of *C3*, *Bf* and *CFH* suggest that multiple alternative pathways with activations by different complement factors may exist in echinoderms. Moreover, the dynamic expression patterns of *C3* and *C3-2* imply that the two pathways may function at different developmental stages [1].

As far as the lectin complement pathway is concerned, its roles in immune response of coelomocytes need to be further explored. As a component of complement system, MBL was found in *A. japonicus* (MBL-AJ) coelomic cavity and reported to play important roles in agglutination and opsonization when inoculated with gram-negative bacteria *Yersinia pseudotuberculosis* [54]. Sequence analysis indicated that *MBL-AJ* contained the conserved carbohydrate-recognizing domain, but lacked the collagen-like domain that was critical for the complement activation [15]. The terminal pathway, which is triggered by C5 complexes (consists of C6, C7, C8 and C9), was not identified in the *A. japonicus* transcriptome as well as in the sea urchin genome [1]. Taken together, C3 and Bf seem to be the primary components of the complement pathway in echinoderms.

### 2.4. Apoptosis

Except for the induction of inflammatory cytokines, LPS may directly cause cell apoptosis (Figure 1). The genes involved in the extrinsic and intrinsic pathways were found in our results and the majority of them (12/14) were significantly induced, such as *caspase-6* (19.3-fold), *cathepsin B* (Cat B) (9.4-fold) and *lysozyme* (5.1-fold) (Table 1). Caspase-6 precursor was identified as an executioner for its role in cleavage of nuclear lamins and apoptosis [55]. However, the active caspase-6 served as an inhibitor to apoptosis [56]. The continuous up-regulation of *caspase-6* at all test time points was observed in this study (Table 1), and further studies on the specific functions of this gene in the immune system of sea cucumber are expected. Cat B, a cysteine protease that is involved in promoting apoptosis, was stringently expressed under normal conditions [57]. Meanwhile, Cat B could induce adjacent cell apoptosis by inducing the release of cytochrome c from mitochondria [58]. After LPS injection, *Cat B* was significantly up-regulated in the coelomocytes of *A. japonicus* (Table 1). The genes with significant expression variations in the apoptosis pathway might be closely involved in the basic host defense against the bacterial infections. Understanding the activating mechanisms of apoptosis pathway by LPS will help us to develop new strategies to reduce the damage caused by bacterial diseases in *A. japonicus*.

### 2.5. Validation of Expression Profiles by qRT-PCR

**Figure 2:**
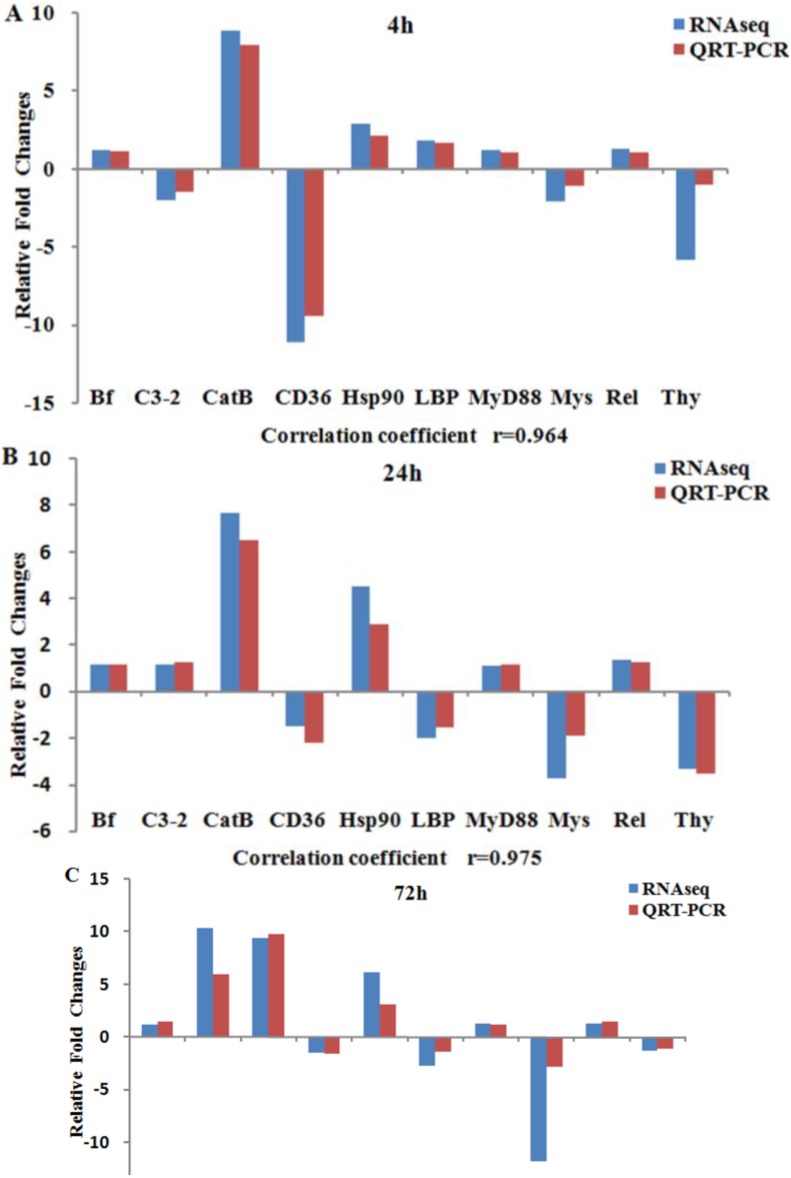
Validation of RNA-Seq results using qRT-PCR. The relative fold changes of 10 genes expressed in *A. japonicus* coelemocytes at 4 h (**A**); 24 h (**B**) and 72 h (**C**) after LPS challenge. Gene abbreviations are: Bf, complement factor B; C3-2, complement component 3-2; Cat B, cathepsin B; CD36, Cluster of differentiation 36; Hsp90, heat shock protein 90; LBP, lipopolysaccharide binding protein; MyD88, myeloid differentiation primary response gene 88; Mys, Myosin; Rel, NF-κB transcription factor Rel; Thy, Thymosin β.

The validation of RNA-Seq results was conducted using qRT-PCR with 10 representative DEGs. The 10 DEGs were selected for their clear background information in the function of immune responses. The comparison between qRT-PCR and RNA-Seq expression analysis is shown in Figure 2. qRT-PCR results were significantly correlated with the results from RNA-Seq at all three time-points (correlation coefficients: 0.88–0.98, *p*-value < 0.05). No consistent bias in expression level was observed for both of the methods (e.g., degree of fold changes are not correlated with method) [59]. Additionally, a single product was amplified with each tested primer pairs by qRT-PCR, indicating the accuracy of contig assembly.

## 3. Experimental Section

### 3.1. Sample Collection and RNA Isolation

Healthy sea cucumbers (average body weight 12.5 g) were kept in aquaria with seawater temperature 18–19 °C, pH 8.0–8.2 and salinity of 3.1% for one week prior to experiment. The sea cucumbers in the treatment group were injected with 500 μL LPS (1 g/L) that dissolved in phosphate buffered saline (PBS) for LPS challenge, while those in the control group were injected with 500 μL PBS instead. At each time point (4, 24 and 72 h) after LPS and PBS injection, 15 individuals from each group were randomly selected and divided into three replicate pools (five individuals each) respectively. The coelomic fluids were prepared using the method reported by Ramírez-Gómez *et al.* [5] with some modifications: By making an incision on the anterior end (tentacled end) of animals, the coelomic fluid was decanted into a clean culture dish in an ice bath and then collected in sterile 1.5 mL centrifuge tubes. Afterward, the coelomocytes in coelomic fluid were pelleted by centrifugation (Labnet Spectrafuge, Woodbridge, NJ, USA) at 4 °C (3500× *g*, 10 min), and then stored in three sterile 1.5 mL centrifuge tubes (five individuals each). All of the coelomocyte samples were frozen in liquid nitrogen immediately and then stored at −80 °C prior to RNA isolation. Total RNA was isolated using the UNIQ-10 Column Total RNA Isolation Kit (Sangon, Shanghai, China) according to the manufacturer’s instructions. The quantity and quality of total RNA extracted from the coelomocytes were measured using the NanoPhotometer (Implen GmbH, Munich, Germany) and agarose gel electrophoresis.

### 3.2. cDNA Library Construction and Transcriptome Sequencing

A total of four cDNA libraries were prepared with the RNA from control and treated groups (4, 24 and 72 h). At each time point, equal amounts of RNA from the three replicates in the treatment group were pooled for library construction. The control library was constructed with the RNA from the replicate pools spanning each of three time points (4, 24, and 72 h). A master pool composed of equal amounts of each replicate control pool was used for RNA-Seq. Poly (A) mRNA purified by Oligo (dT) must be fragmented before double strand cDNA synthesis. The first strand cDNA was synthesized with the random hexamer primer and the second-strand cDNA was synthesized using RNaseH and DNA polymerase I. After the end repair process, addition of “A” base and ligation of sequencing adapters, the suitable fragments purified by agarose gel electrophoresis were chosen to construct the cDNA libraries. High throughput sequencing of the libraries was carried out on the Illumina HiSeq™ 2000 platform to generate100-bp paired-end reads (BGI, Shenzhen, China).

### 3.3. Analysis of Differentially Expressed Genes

Before the assembly, the raw data was trimmed to remove low quality reads. The clean short reads from this study and the existing 454 reads and ESTs from GenBank were used in the *de novo* transcriptome assembly [35,37]. GO terms were analyzed by Blast2GO (Instituto Valenciano de Investigaciones Agrarias, Moncada, Spain) [60]. Pathway analysis was conducted based on the KEGG database to give an overview of regulation networks. The gene expression levels were assessed by RPKM (Reads Per kb per Million reads) method, which eliminated the influence of different gene length and sequencing level on the calculation of gene expression. To identify the DEGs in the coelomocytes of *A. japonicus* at different time points after LPS challenge, a rigorous algorithm was developed for statistical analysis according to “The significance of digital gene expression profiles” [61]. False Discovery Rate (FDR) was used to correct for *p*-value [62]. When we got FDR, the fold changes between two samples were calculated by ratio of RPKMs. To judge the significance in gene expression difference, we set “FDR ≤ 0.001 and the absolute value of log2Ratio ≥ 1” as the threshold [63]. The lager ratio indicates the lager difference of the expression level between the two samples. Above analysis was performed on the RNA-Seq module and the expression analysis module in CLC Genomics Workbench (CLC bio, Aarhus, Denmark).

### 3.4. Expression Validation Using qRT-PCR

In order to validate the reliability of RNA-Seq data, qRT-PCR of 10 differentially expressed immune-related genes was performed on the Mx3005p™ detection system (Agilent Stratagene, Santa Clara, CA, USA). Total RNA from control and tested samples used in RNA-Seq was reverse-transcribed into cDNA templates with the PrimeScript™ RT reagent Kit (TaKaRa, Otsu, Japan) according to the manufacturer’s instruction. The reaction program consists of two steps: 37 °C for 15 min and then 85 °C for 5 s. All the fluorescence quantitative primers were designed using Primer 5.0 software according to rigorous criteria. The primer information was provided in Table 2. The cytochrome b (Cytb) gene was chosen as the reference gene [14]. Optimal primer pairs were examined by checking the melting curve at the end of each PCR reaction to confirm the specificity of PCR product. The qRT-PCR amplification was conducted in a volume of 20 μL containing 10 μL of 2× SYBR Premix Ex Taq™ II (*Tli* RNaseH Plus, TaKaRa, Otsu, Japan), 0.4 μL of ROX Reference Dye II, 2 μL of cDNA template, and 0.4 μM of each primer according to the introduction of SYBR Premix Ex Taq™ II Kit (*Tli* RNaseH Plus). The thermal cycling profile of qRT-PCR program was 95 °C for 30 s, followed by 40 cycles of 95 °C for 10 s, 56 °C for 25 s and 72 °C for 25 s. The expression levels of target immune genes were normalized by the reference *Cytb* gene. Relative Expression Software Tool 384 v.2 (REST) (Technical University of Munich, Munich, Germany) [64] was used to calculate the expression differences between control and LPS challenge groups. The relative expression levels were assessed in group mean by pair wise fixed reallocation randomization test. Each measurement was performed in triplicate. The correlation analysis between RNA-Seq and qRT-PCR results was carried out with SPSS17.0 software (IBM Corp., Armonk, NY, USA). A *p* < 0.05 was considered as statistically significant.

**Table 2:** Primers used for libraries construction and qRT-PCR.

Gene	Primer Sequence (5'-3')
*Sequencing adaptors*	5-primer: AGATCGGAAGAGCGTCGTGTAGGGAAAGAGTGTA
	3-primer: AGATCGGAAGAGCACACGTCTGAACTCCAGTCAC
*CD36*	CD36-F: ATTCTTAAAGCCAGCCACA
	CD36-R: AGTCGTTAGCCGAAGCACC
*Complement component 3-2*	C32-F: CTCTCGTGAGTTCTGGC TCAG
	C32-R: GCAGCCACTGTTACCATCGCGGA
*Complement factor B*	Bf-F: ATTATCTCGCAACAGCGATCC
	Bf-R: GGGCAACCACACCGGCTTCTCCA
*Cytochrome b*	Cytb-F: TGAGCCGCAACAGTAATC
	Cytb-R: AAGGGAAAAGGAAGTGAAAG
*Heat shock protein 90*	Hsp90-F: TATGAAAGCCTGACAGACGCAAGC
	Hsp90-R: TAACGCAGAGTAAAAGCCAACACC
*Lipopolysaccharide-binding protein*	LBP-F: AGAAGGGAAATCATACAGAGGCACC
	LBP-R: TAGCAACATAGTCAGTCATCCACAT
*Myosin V*	Mys-F: GGGGTGGTCGTCTGATTTGC
	Mys-R: AAGGTGATTTGAGGAGCGGTA
*Myeloid differentiation primary response gene 88*	MyD88-F: CCGATGTAGGAGGATGGTAGTAG
	MyD88-R: CACAGTAAGGTGCTGAAGAATGC
*NF-κB transcription factor Rel*	Rel-F: TGCGAAGCCACATCCATT
	Rel-R: AGGGCATCCTTTAAGTCAGC
*Thymosin β*	Thy-F: GAGCAGGAGAAAGCAACATAG
	Thy-R: GAACAAAACAAGCACCCATT

## 4. Conclusions

In this study, Illumina RNA-Seq technology was used to characterize the dynamic expression profiles of genes in *A. japonicus* coelemocytes after LPS challenge. One hundred and seven immune-related DEGs were summarized and classified into four groups (pathogen recognition, reorganization of cytoskeleton, inflammation and apoptosis) according to their functions in response to pathogen invasion. The candidates with novel expression patterns may be useful in the identification of potential resistance markers related to bacterial diseases such as SUS in *A. japonicus*.
